# Discourses of Nature in New Perceptions of the Natural Landscape in Southern Chile

**DOI:** 10.3389/fpsyg.2018.01177

**Published:** 2018-07-17

**Authors:** Enrique Aliste, Mauricio Folchi, Andrés Núñez

**Affiliations:** ^1^Department of Geography, University of Chile, Santiago, Chile; ^2^Department of Historical Sciences, University of Chile, Santiago, Chile; ^3^Institute of Geography, Pontifical Catholic University of Chile, Santiago, Chile

**Keywords:** nature, green imaginary, forestry plantations, eco-tourism, Patagonia, natural landscape

## Abstract

Landscapes are shaped over time by the changing imaginaries that result from new representations of nature and the value associated with it. This paper discusses the evolving discourses which have shaped the perception of the landscape in two socially and ecologically significant contexts in Chile. The first is the central-southern region of the country, a large portion of which is now devoted to commercial forestry plantations. The second is the Patagonia-Aysén region, where since the 1990s, colonization of a land defined by a tradition of livestock rearing has evolved into a process epitomized by the slogan “Aysén, Life Reserve.” The representation that was made of central-southern Chile in the 50' and 60' as a deforested and degraded land was the justification for promoting a new form of land occupation: the monoculture forest, designed and executed by a specific law. Forty years on from the passing of this law, the plantations of central-southern Chile have undergone a process of naturalization. In this case, the exaltation of nature has been permanent (before and after the changes doing by this law). The only thing that changes is the definition of nature, which ended up including forest plantations. That is, discourses influence perceptions and these lead to new practices in the study area and beyond. In Patagonia-Aysén, by the other side, there has been a marked shift in the rhetoric surrounding land. This has been particularly noticeable in the case of government bodies and private ecotourism companies, which have constructed an imaginary in line with a new model of economic development for the area. In a break with tradition, both the public and private sectors are beginning to shift their investment away from agricultural and livestock exploitation and toward ecotourism and conservation projects. In both cases, we analyse the manner in which transformations in perceptions and representations of landscape bring about new forms of land use, and how new focuses of value and social interest, forged within wider environmental discourses, have brought with them unexpected social consequences, like depopulation, economic transformations, cultural changes, etc. Thus, the aim of this work is to expose and discuss the reality and scope of new green discourses and their influence on the perception of natural landscapes in the Chilean neoliberal context.

## Introduction

What relevance do discourses of nature have to the notion of landscape, and, in particular, natural landscapes? In recent years, a variety of notions and perceptions have highlighted how the concept of *natural* has become an increasingly dominant, desirable and important theme, evolving into a contemporary ideal from which normative and organizational criteria of territory and landscape have emerged. The discussion has been enlivened by widespread debate on the subject, and emphasis is put on the idea of understanding the political nature of the topic, rather than viewing it simply as a neutral element. To achieve this, there is a need to identify the conditions which define the characteristics and intentions which ultimately lead to the creation of territories (Castree, 1995, 2008, 2014; Harvey, 1996; Smith, 2008; Swyngedouw, 2015). While the aim of this article is not to enter into the discussion as such, it does propose to explore the extent to which certain discursive practices contribute to interpretation of processes of landscape production in two specific contexts in Chile, and how this influence has helped to shape certain territorial processes.

As part of this, we must consider that, since the beginning of the twenty-first century in particular, the environmental crisis and the various discourses surrounding the need for societal respect for and harmony with our natural surroundings have become a maxim affecting public policy, international relations, politics, and numerous issues to do with society's growing need to regain a notion of landscape capable of addressing the concerns of our age (Lascoumes, 1994). In the words of Corbin:

“[…] Political preoccupation in the West with preserving, observing and restoring landscapes has arisen only recently […]. This is the result of the diverse ways of valuing space. Preservation of the landscape, which is a growing concern, simultaneously represents a source of enjoyment, a trigger for conflict, an instrument of power, and a matter of identity. The idea of environment, ecological concern, and the desire for nature all contribute to the growing notion of landscape”. (Corbin, 2001, p. 149).

We need, therefore, to explore in greater detail this concept of *natural*, and, in particular, to attempt to identify what is actually meant by the notion of nature as invoked by such discourses. To do so, we should consider nature as defined by Castree (2008) as we explore why non-human phenomena have become increasingly neoliberalized across different parts of the world, and how the use of certain management concepts based on this rationale (governance, value attribution, services, etc.) help to promote a style of discourse capable of redefining the way in which we perceive nature.

The present article seeks to analyse transformations in landscape—in particular those changes to how landscape is perceived—and comprises a continuation of the discussion surrounding the idea of nature, discourses on the subject, and the peculiarities of each type of landscape transformation. Above all, the article strives from a historical-geographical standpoint to identify the influence that changing discourses have had over time on the way landscape is perceived, along with some of the effects that they have brought about.

In the words of Nogué:

“Interpretations of the landscape, and indeed the landscape itself, reflect a particular approach to organising and experiencing the visual order of geographical objects in the territory. Thus, landscape contributes to the naturalisation and normalisation of social relations with the established territorial order” (Nogué, 2009, p. 12).

While the concept of nature is relevant in this context, it should be considered as an element that serves to emphasize the conditions that produce a particular type of landscape. Indeed, there are actions which, driven by certain imaginaries, inject into the different perceptions of the landscape content capable of influencing the way in which society organizes its knowledge, ideologies and different forms of cultural production, that in turn impact upon the landscape and its use.

The present work addresses two cases which assist not only in questioning the status of this idea of nature and the landscape that it shapes, but also seeks to explore specific experiences in order to analyse the possibility of opening a discussion concerning two changes of direction in territorial production which are gaining momentum today. These are transformative discourses on the environment and on social and cultural conditions within the territory, and they make use of certain discourses of nature. In short, the work seeks to question the concept of “natural landscape” as a social and political element used to contribute to the redefinition and reorganization of territory in Chile.

We will cover two cases. Firstly, the land owned by forestry companies along the coastal regions of central-southern Chile. These estates have been consolidated with the help of policies implemented primarily during the second half of the twentieth century, and today form vast extensions of commercial monoculture forestry plantations. These constitute the natural basis of the landscape for major sections of the local population, and define territorial identity. The second and contrasting case is that of the so-called “neo-colonization” or “eco-colonization” taking place in recent years, which is generating renewed appreciation of the value of the pristine, wild nature of Chilean Patagonia. This phenomenon has shifted focus in the Aysén region away from the traditional activities of inhabitants, who for more than a century have been concerned with livestock rearing—a cultural landscape which today is practically invisible in the region—to be replaced by major real estate businesses involved in conservation and ecotourism.

**Figure 1:**
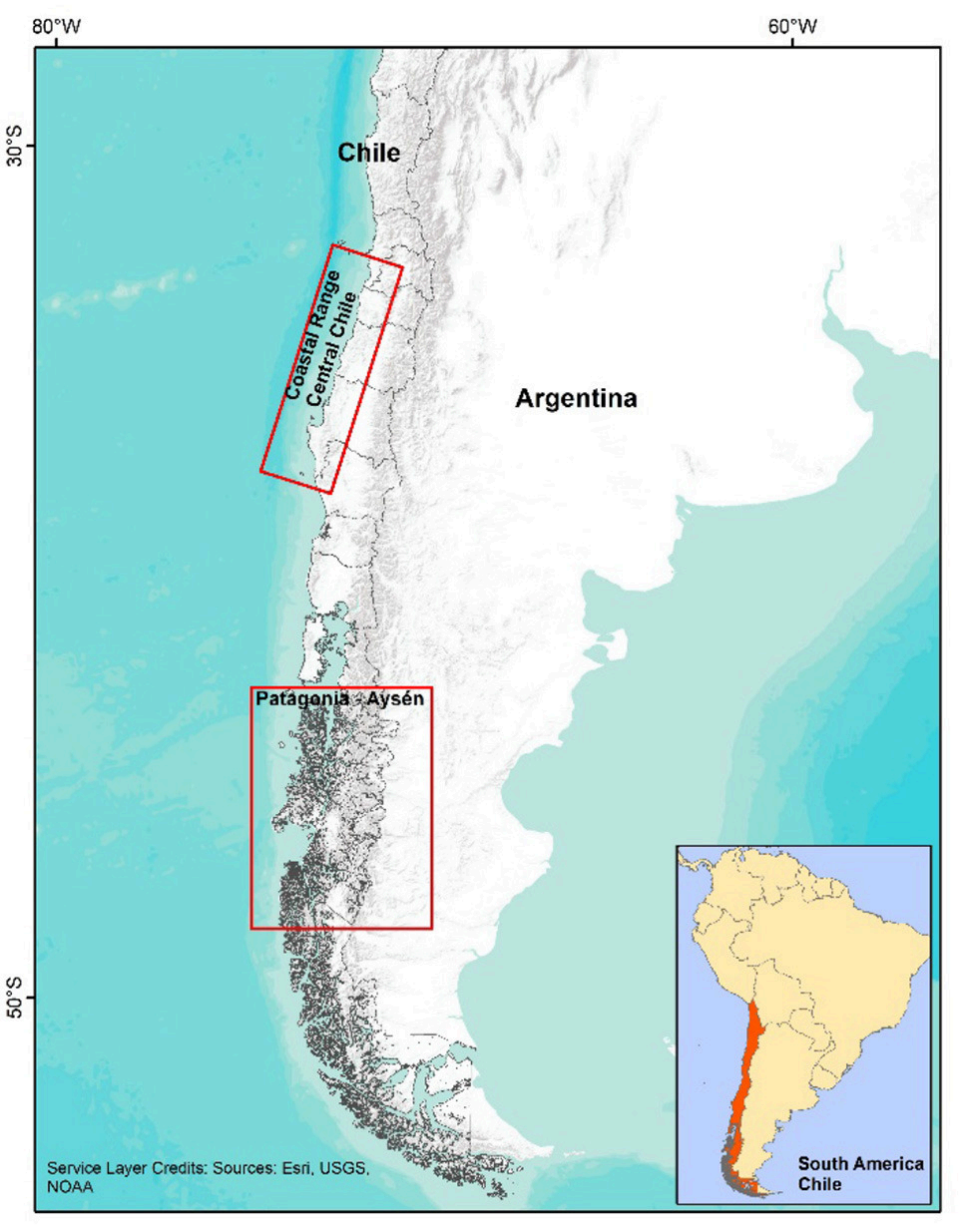
Study areas. Source: Compiled by the authors based on ESRI, USGS, NOAA.

The study area is presented in Figure 1.

Based on these two case studies we hope to promote discussion and reflection in terms of the extent to which and the way in which the natural landscape is perceived, and identify some practical consequences of these perceptions.

## Materials and methods

Reconstruction of the history and discourse of the landscape of the *Cordillera de la Costa* (Coastal Mountain Range)^1^ and of related forestation policies is carried out by means of a documentary and literature analysis. We analyse the contents of documentation created by institutions and bodies involved in the construction of this landscape between the 1930s and the present day, including that of the Forestry Institute (INFOR), the National Forest Corporation (CONAF), and the Ministry of Agriculture. This analysis is complemented by fieldwork and open interviews with inhabitants of the areas subject to forestation. Additionally, we perform a reconstruction and analysis of statistical information pertaining to the forestry sector from 1970 to the present.

For the Patagonia-Aysén case study, a series of open interviews were conducted in the field. Interviews focused on traditional inhabitants who have witnessed the territorial transformations of the last 40 or 50 years, and took the form of open, guided conversations in a free format. The intention was that these dialogues would provide information concerning processes of land use, recognition of value, and identification of transformations, within a qualitative framework and with a firm phenomenological base. Meanwhile, we conducted a study of sources such as official registers and documents from public entities including the former Ministry of Land and Colonization, and the Coyhaique and Cochrane Land Registry, which has permitted, through use of spatial analysis techniques, the creation of cartographic representations of transformations and changes in land ownership.

In both case, the inspiration has the geo-historical approach, that its consist in the observation of the transformations of the existing territories, that can be read in long cycles hidden behind shorter cycles that directly influence the current organization of the territories (Musset, 2009).

## Nature and landscape: an environmental history for understanding territorial dynamics

The notion of nature is addressed by innumerable schools and traditions which, from a philosophical perspective, offer a variety of forms, fields and ideas for exploring the concept. For now, bearing in mind this broad range of possibilities and recognizing the enormous scale of the concept of nature, it should be noted that our focus will be on its modern interpretation, and will be kept brief. The intention is to support the idea of natural landscape being in a dialogue with the notion of the environment. We adopt Foucault's principle that the threshold of modernity can be placed precisely at this point of separation between nature and human nature (Foucault, 1999).

The history of landscapes and of the environment can be understood as a process of cultural history which in turn evolves into an environmental history. The proposal for understanding an aesthetic pattern—its ecological value; its social, economic or value-based contribution—is built on the relationship which society develops with its surroundings and its time (Ingold, 2000; Corbin, 2001; Delort and Walter, 2001; Walter, 2004; Lugynbühl, 2008). This requires that an environmental history be understood as a two-way process that identifies both the impact of the environment on society and its attributes, and the degree of influence that society in turn exerts on environmental transformations (Worster, 1988; O'Connor, 2001). As such, the study of environmental history is not only desirable, but should be considered an indispensable tool for analysis, serving to gain a deeper appreciation of the complexity involved in understanding concepts such as landscape, territory and nature. Notions such as Fernand Braudel's *longue durée* (long term) explore the link between space and time, identifying an inextricable relationship between the processes that bring to life certain social, cultural, economic and political characteristics of territories (Braudel, 1969; Cunill, 1995, 1999; Aliste and Musset, 2014). An environmental history should form part of an integrated strategy in which the study of social, cultural, political and economic processes can be effectively articulated with a comprehension of spatial and environmental transformations, such that an understanding may be gained of the ecological dimension of these processes (Aliste, 2010; Aliste and Musset, 2014). Thus, concepts such as landscape, space, territory, environment and sustainability must be subjected to scrutiny in order to provide context for possible temporal or spatial interpretations, and so construct an image of what a landscape means to the society of which it is a part, and then, the importance of discourses, imaginaries and representations for understand these living spaces (Di Méo, 1991; Berque, 1994; Frémont, 1999; Ingold, 2000; Bialasiewicz, 2006).

H. Lefebvre makes an interesting point on this subject by underlining the idea of representations. The author claims that the notion of nature is a nostalgia, and that it lends itself to manipulation (Lefebvre, 1983). How and why do these concepts keep coming back into the discussion, even today? It would seem that they resurface in the context of new (if they can be called that) ideas about protection, conservation and restoration of nature or the natural world. What is this nature that lies at the heart of this discussion? Swyngedouw (2015) and Castree (2005, 2008, 2014) claim that nature does not exist, and that it is instead a product of society which has contributed strongly to the consolidation of a neoliberal approach to our relationship with our surroundings, strongly supported by notions such as governance, management, value attribution and services. These notions have become more commonplace since the advent of environmental discourses, or of those which promote the concept of landscape with a natural connotation. The authors stress the need to consider these concepts along with their political associations.

There is therefore great relevance in the exploration of spatial production (Lefebvre, 1974), and especially of the way in which landscapes are transformed not only through material modifications, but through changes occurring within society. For example, one interesting point of view is developed by Menatti (2017) in terms of conceived the landscape coming from common good to a human right. The latter ascribe meaning and value to a landscape according to new knowledge structures, new value frameworks, or certain social agreements defined by contingent norms. By the same logic, some situations provide an opportunity to exert power or impose social segregation (Roger, 1995; Escobar, 2000; Porto-Gonçalves, 2001, 2006; Neumann, 2003; Himley, 2008; Marcel, 2008; Aliste, 2010; Miles, 2010; Carman, 2011; Grove, 2014; Latta, 2014; Poe et al., 2014).

Thus, by reflecting on the dynamics of landscape and on the influence that perception exerts on geographical definitions of the same, an exploration of the cases of the forestry plantations of central-southern Chile's Coastal Range, and the idea of nature in its purified form in Chilean Patagonia may provide for further reflection on this subject.

### The coastal mountain range: a represented, disputed, and occupied landscape

**Figure 2:**
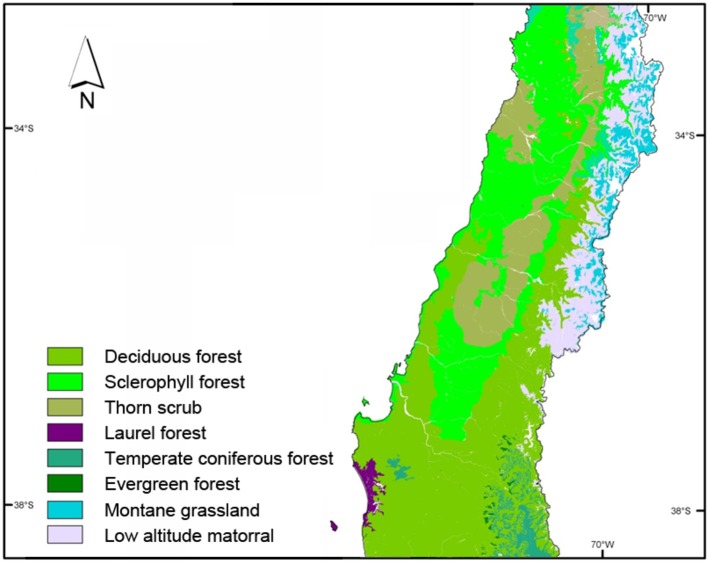
Natural vegetation in central-southern Chile. Source: Luebert and Pliscoff (2006).

The *Cordillera de la Costa*, or Coastal Mountain Range, is one of the four relief macroforms of central-southern Chile, constituting a chain of mountains running the length of the country Its highest peaks (over 2,000 m) are found at the latitude of Santiago, and the range lowers toward the south. Around the city of Concepción it becomes an irregular plateau with an average height of 500 m. South of the Biobío River the range begins to rise, reaching 1,000 m before losing height again. The soil, a dark reddish brown in color, was formed from granite and reaches a depth of up to 120 cm (Luzio, 2010). The climate becomes drier toward the North and more humid to the South. As a result, the soils have developed a covering of vegetation dominated by thorn scrub (*Acacia*) and Mediterranean sclerophyllous shrubland (comprising evergreen species) in northern areas, and deciduous forest (*Nothofagus*) in the South (see Figure 2). This landscape remained consistent until the nineteenth century, when expansion of the agricultural frontier away from the Central Valley led to the adoption of these lands for cultivation of crops, livestock rearing and charcoal burning (Donoso, 1998).

Toward the end of the 1930s, there emerged a generation of agronomists concerned with the conservation of natural resources, and one of their primary worries was the problem of soil erosion. In May 1940, one of their number, Manuel Elgueta, attended the Eighth American Scientific Congress hosted in Washington D.C. He participated in the section dedicated to “Agriculture and Conservation,” led by the North American soil conservation expert, Hugh Hammond Bennett, with a presentation entitled “Soil Erosion in Chile.” During his talk, Elgueta (1941, p. 122) described the problem of soil erosion in Chile as follows:

“In the central region of Chile there is much erosion in the arid coastal sections where it is not irrigated. During the long, dry summers all vegetation dies out and the slopes of the coast mountains are exposed to the intense rains of the winter months. Erosion is aggravated in some places where wheat is grown. In the wheat lands the soil is plowed clean and is left exposed to heavy winter rains. Wheat is grown every other year. In the fall when seeds are plowed under, another winter adds its deteriorating force to the erosion process. Gullies are characteristic of the coastal slopes of Chile. These gullies tell the story of erosion in this region.”

His description of the southern part of the country was even more stark:

“The hilly country, the enormous amount of rains, and the type of agriculture have brought about one of the most serious erosion problems of which I know. The first settlers —the pioneers who cleared the forest for agriculture— destroyed the trees by burning them. On the land cleared by the fire, they sowed their wheat. The first plantings brought high yields. Over a period of years this region was a rich and prosperous wheat country. But, unfortunately, wheat was the only crop. The type of farming consisted of plowing the land clean one year and sowing the seed the next year. Erosion began to carry away the topsoil […] In a little more than fifty years, all the topsoil has been washed off. What we see when we travel through the province today is a reddish soil —really the subsoil.” (Elgueta, 1941, p. 123).

**Figure 3:**
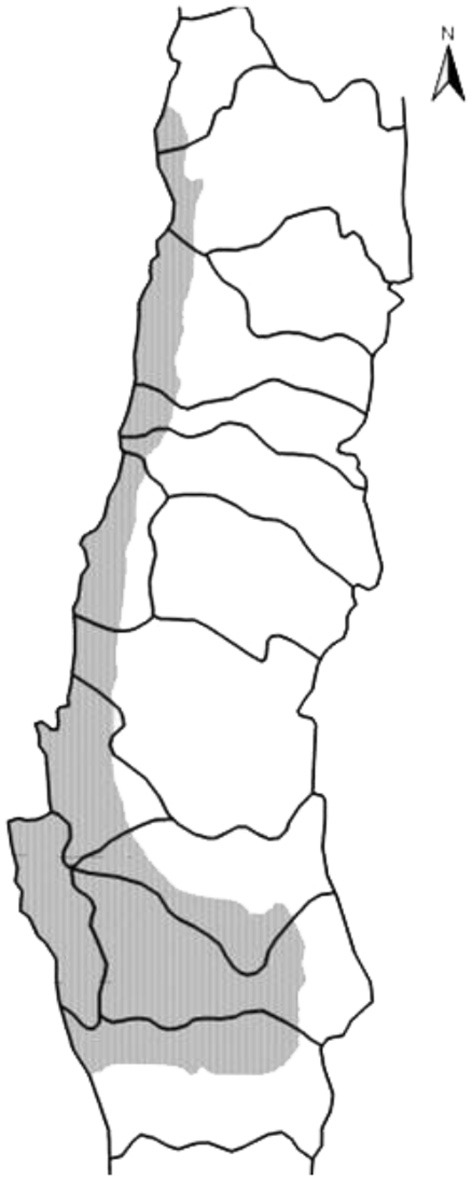
Eroded area (1943). Source: Bianchi (1947).

This initial evaluation was followed by several studies from the same group of agronomists, who held posts at the Ministry of Agriculture and other State departments (Elgueta and Jirkal, 1943; Rodríguez and Suarez, 1946; Bianchi, 1947; Rodríguez and Díaz, 1951). These studies served to reveal the magnitude of the problem affecting vast tracts of the country (see Figure 3), analyzed the causes—which were both economic and social—and sought for solutions. These included modifications to exploitation methods, the implementation of soil defence projects, the introduction of new cultivation techniques, the improvement of pasture and livestock management, and, finally, the reforestation of “certain areas which, due to soil conditions, must be dedicated exclusively to this purpose, whether this be because economically they are unsuitable for continued cultivation, or because the formation of forests is vital to conservation of the soil (Ministry of Agriculture, 1945, p. 223). It should be noted that, up to this point, forestation had been considered as just one of many measures that attempted not only to deal with the problem, but to combat its causes.

Anxious about the situation, this generation of experts saw as concerning the possibility that these degraded or threatened soils could be forested indiscriminately. In 1946, Manuel Rodríguez, head of the Ministry of Agriculture's Soil Conservation department, criticized the forestation (with pine and eucalyptus) which had occurred across some parts of the Coastal Range:

“We must draw attention to the danger posed by forestation of agricultural fields. Under no circumstances should they be withdrawn from cultivation, as this act would remove valuable soils from the country's already diminished agricultural land. This is yet more inconceivable when we consider the thousands of hectares unsuitable for any kind of use save forestry, and which must be planted as soon as possible in order that they not become completely useless. A typical example of this flawed policy is the foresting of thousands of hectares of rolling hills on the Santiago coastline near to the towns of Llolleo and Cartagena, whose soils are perfect for cereal production and the formation of rain-fed pasture” (Rodríguez and Suarez, 1946, p. 44).

It was in this context that timber companies began to make their discourses heard, identifying the problem of soil degradation and proposing forestation as the best—if not the only—strategy for resolving the problem.

In 1951 they launched a channel for promoting their ideas and interests: the *Revista Forestal Chilena* (Chilean Forestry Review). The publication made the case for the country's “forestry vocation”, and emphasized the need for the industry to be developed. One year on, the timber producers' trade association *Corporación de la Madera* (CORMA) was formed. Among its primary initiatives was the proposal put to the government for a large-scale programme of forestation to cover a minimum of 100,000 hectares per year, which would make it possible to:

“put to use once again vast areas which currently lie abandoned, and bring to an end the threat of destruction that hangs over many valuable arable soils. Given the enormous capitalization and wealth which would thus be generated, it can only be hoped that future men of the government will recognise the transcendental importance that such a programme of forestation would have for the future of the country, and put their full support behind the creation of vast extensions of ‘Green Gold”'^2^.

Besides aligning itself with the developmentalist discourse that predominated in the country at the time, CORMA introduced the (eminently determinist) idea that Chile was “a forest country”: that this was its nature, and that it was this activity which would be the source of its wealth and prosperity. This definition of a natural vocation considered the exploitation not only of “jungles” (native forest), but also of “artificial forests.” In 1954, CORMA urged the government to abandon its “passivity” in the face of the forestry sector's problems, and implement measures necessary to encourage its development. In doing so:

“the Chilean economy will steam towards a prosperity visible in nature itself, granting the country vast jungles, and extraordinary climatic and soil conditions to preserve them as an inexhaustible source of wealth for its inhabitants”^3^.

The president of the trade association, Julián Echavarri, expressed himself in similar terms in 1956, adding a certain nationalist flare to the forestry discourse, maintaining that:

“There is no activity more closely bound up with the very essence of the fatherland than those activities which, directly or indirectly, have to do with forests. Trees are not simply a commercial product: their roots are buried deep in the heart of the nation. They are linked to our homes, to the maintenance of our agricultural land, to the balance of our climate, to the flow of the rivers, to the power of our hydroelectric power stations, and indeed to every facet of Chilean life”^4^.

The Chilean State's concern over forests had begun in the 1920s. At the behest of the first silviculture expert to step onto Chilean soil—German naturalist Federico Albert—the country's first Forest Law was passed in 1925, with the objective of structuring the use of national forests and promoting the planting of new ones (Camus, 2003; Ramírez, 2007; Folchi, 2015). To this end, a fiscal system was put in place to subsidize the cost of State nurseries, and grants were offered for those private landowners and local councils that wished to be involved in forestation. In addition, all forestry land was made exempt from paying “all fiscal and local taxes”^5^.

This first forestation policy was not particularly successful due to the limited resources made available for it. In 1936, it was estimated that “artificial forests” covered a total area of 90,548 hectares (Ministry of Agriculture, 1945, p. 32). From the 1940s onward, the Chilean State increased its promotion of forestry, but not as a means of soil conservation. Rather, it was in response to the imminent depletion of natural timber forests which had been devastated in the past (Otero, 2006; Folchi, 2015), and because of the need for timber for urban expansion and as a primary material for the growing pulp industry. The latter formed part of the import substitution industrialization (ISI) development model being promoted by the State. The fundamental instruments of this policy were the expansion of State nurseries and the granting of loans for the planting of the fast-growing tree species *Eucalyptus spp*. and *Pinus radiata*, the creation of the country's first pulp mill (1959) and the founding of a dedicated technical body, the Forestry Institute in 1961. As a result of these measures, by the mid-1960s, the total forested area in the country's central-southern region had increased to 277,944 hectares.^6^

In 1965, the State became further involved in the forestation process, this time with the explicit intention of protecting degraded soils. That year, the government designed the ambitious National Reforestation Plan which proposed to plant five million hectares by the year 2000. This area equated to the soils “suitable for forestry” that had been affected by erosion. The Plan never went ahead, but many other more focused forestation plans did materialize on both privately owned and State land the length of the country, all of which were overseen by the Reforestation Corporation in 1970. It is estimated that by 1973, the total forested area had reached 478,918 hectares. President of the Republic Eduardo Frei Montalba (1964–1970) explained the motives for this forestation effort during his last message to Congress:

“The foremost of our national riches are our earth and water […]. Is it not true that erosion is destroying major agricultural regions, and that we look on, unconcerned, as millions of tonnes of topsoil are swept out to sea? […]. With our trees burned and destroyed, particularly in river basins, these rivers now scour the living earth, losing the regularity of their courses or sweeping over the land in destructive torrents […]. How can we ignore that across extensions of land spanning more than a thousand kilometres, where once there was a covering of native vegetation, there now remains only naked rock? […]. If I still have any authority to speak to my fellow citizens, it would be to point out this problem, and say that we, as a nation, must strive to resolve it” (Frei, 1970, p. 87–81).

**Graph 1:**
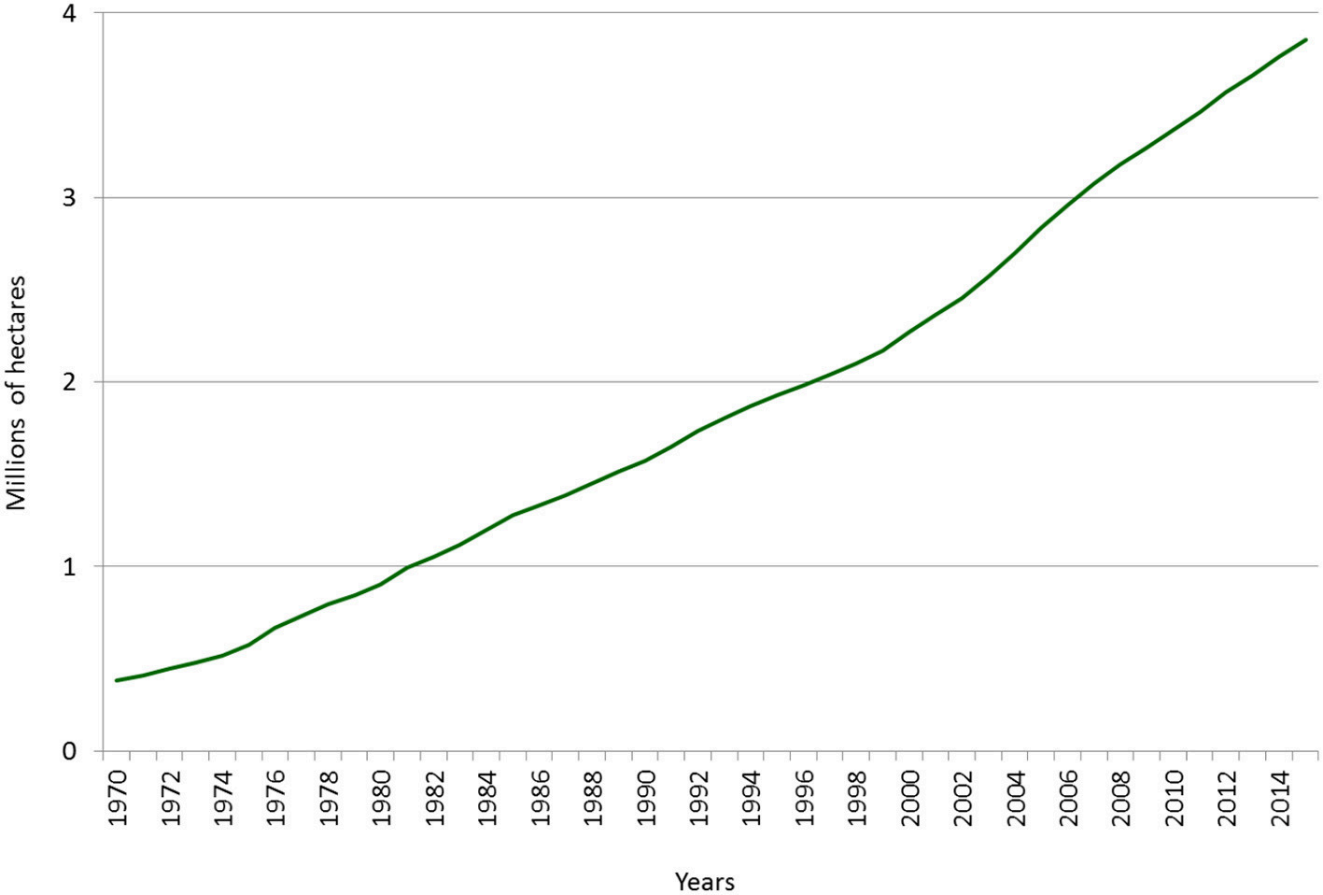
Planted area, 1970-2014 (hectares). Source: INFOR. Forestry statistics. Note: Calculated based on the annual register of newly planted area.

**Graph 2:**
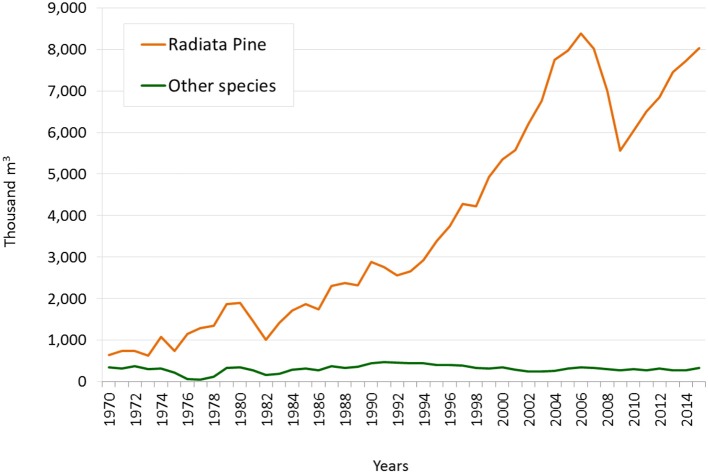
Sawn timber production, 1970–2014 (thousand m^3^). Source: INE until 1973; INFOR from 1974 onwards.

Later, with the advent of the military dictatorship (1973–1989), a new economic development strategy was adopted based on expansion of the export sector. Meanwhile, the State's role in the economy was reduced, with private initiative being encouraged to take its place. Both principles were applied successfully to the development of the forestry industry by means of an aggressive development policy embodied by Decree Law N. 701 of 1974, which established a series of forestation initiatives. The most important of these was a 75% subsidy on the cost of forestation. At the same time, development loans were offered by the Central Bank to stimulate private forestry, as well as exemption from land tax and a guarantee of protection against expropriation of land designated “suitable primarily for forestry.” Meanwhile, State-owned forested areas were passed into the hands of forestry companies. This situation marked a new period in the sector's history, defined by accelerated growth and an absence of direct State participation. The area planted with exotic species went from 576 thousand hectares in 1975 to almost 3.7 million hectares in 2014 (see Graph 1). The industry's physical production grew proportionally. In 1975, sawn timber production was at 976,000 m^3^. By 2014 it had reached almost eight million cubic metres (see Graph 2).

**Figure 4:**
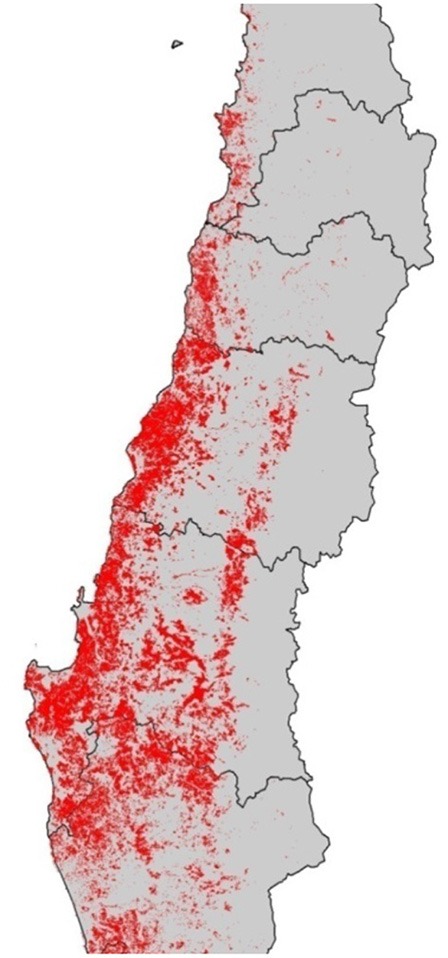
Forested area (2011). Source: Compiled by the authors.

The forestry plantations were concentrated primarily in the area surrounding the Coastal Range and between the country's sixth and ninth regions, based on the assertion that these were soils which “needed” to be forested (see Figure 4). Until the 1960s, these lands had been used for agriculture and livestock, accommodating a significant rural population and defining a landscape which combined rural tradition with coastal countryside. Change of soil use posed great difficulty for the continuation of agricultural activities, and was accompanied by scarce to non-existent compensation in terms of the contribution of the plantations to the rural economy. On the contrary, the situation forced the displacement of small rural producers from forested territories. Meanwhile, forestry expansion also had significant environmental consequences, starting with the replacement of native vegetation covering—according to the most conservative estimates-−262,967 hectares (Prado, 2015, p. 69). This was on top of the diminished availability of water in river basins forested with fast growing exotic species, and the deterioration of soils resulting from bad forest management practices.

**Figure 5:**
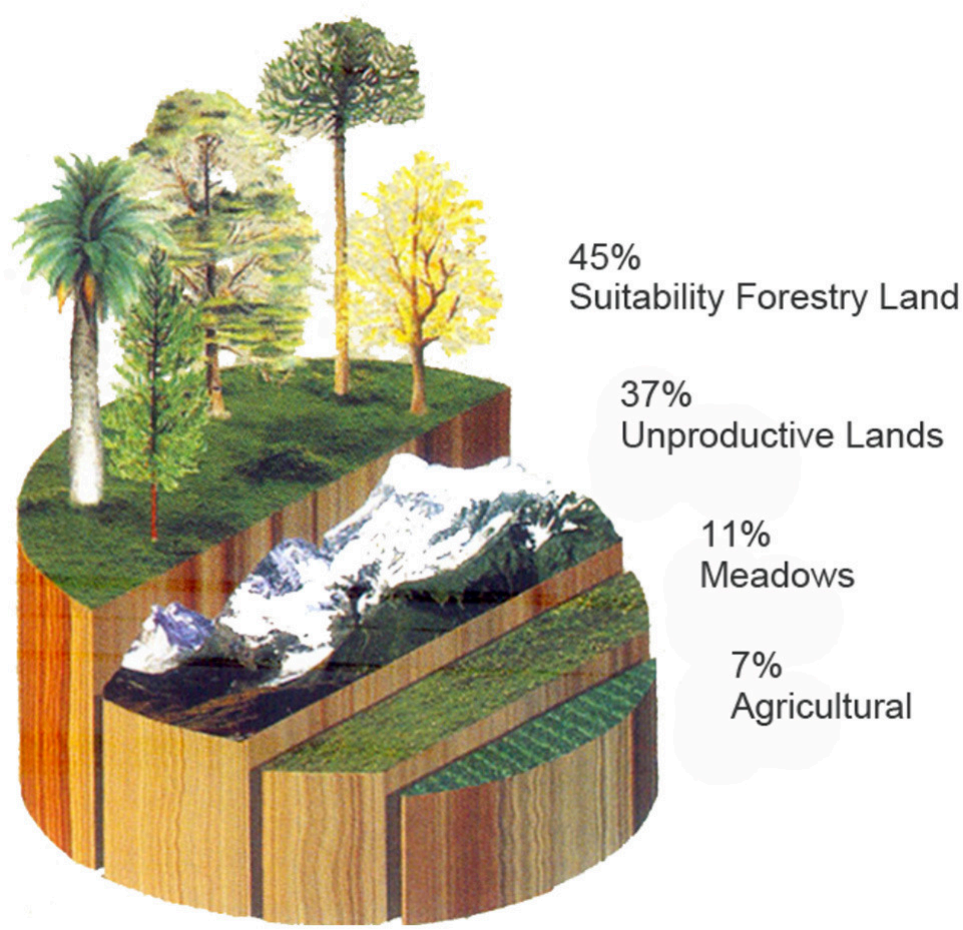
Soil distribution according to CORMA. Source: Corporación Chilena de la Madera (1999). *Forests of Chile*.

**Figure 6:**
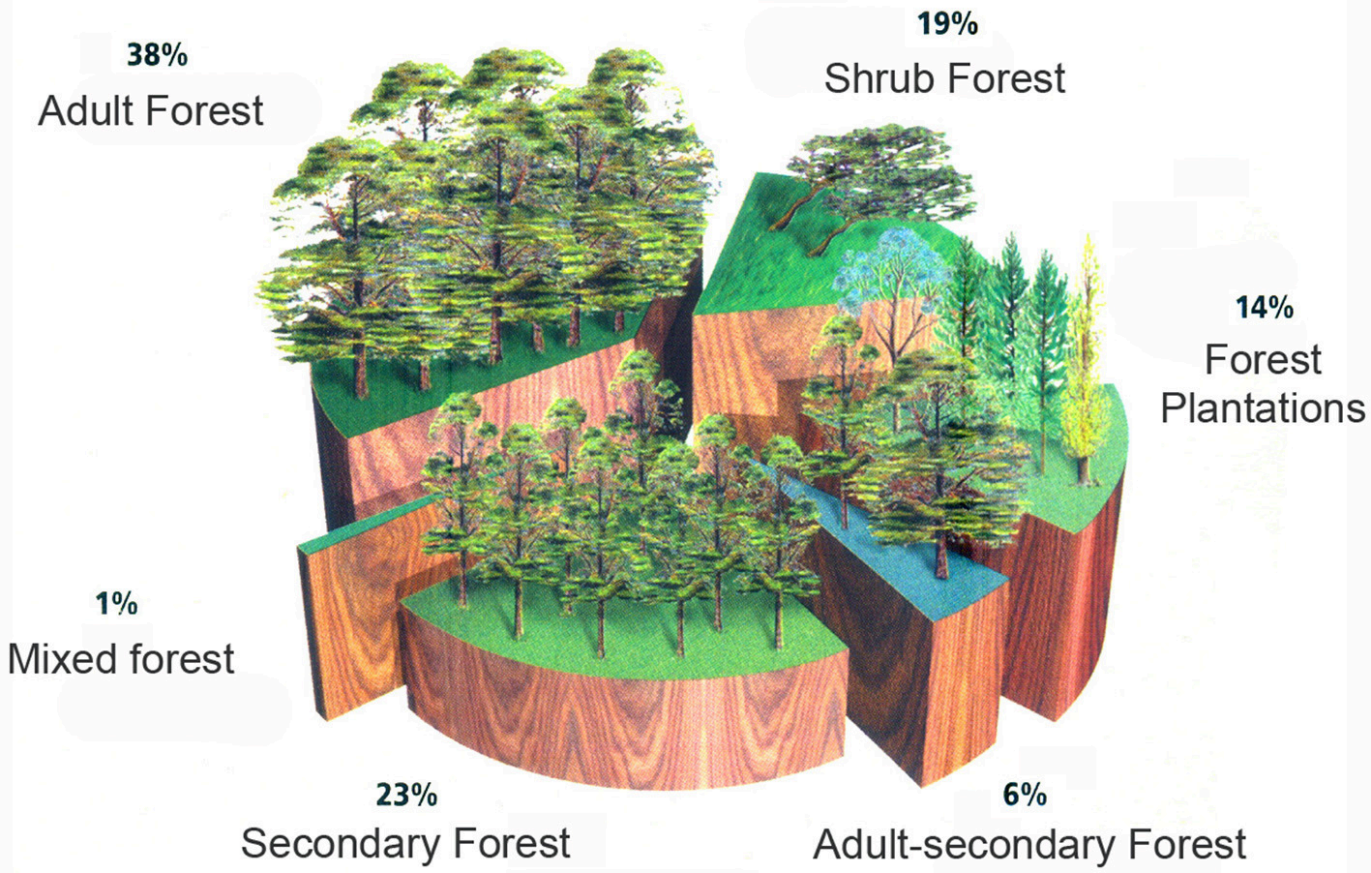
Forest distribution according to CORMA. Source: Corporación Chilena de la Madera (1999). *Forests of Chile*.

In response to these criticisms, the forestry companies have persevered in their construction of a natural imaginary to do with the expansion of forestry activity. Their discourse emphasizes first and foremost that Chile is a “forest country”, in which the majority of soils are suitable for forestry (see Figure 5). They then turn to history, arguing that a major portion of forests would have been removed to make way for agricultural activities, and that associated bad practices would eventually have destroyed the soils beneath. Following this disaster, the forestry companies would have arrived to plant trees where Nature had put them originally, incorporating these “artificial forests” into the country's marvelous “forest heritage” (see Figure 6)^7^.

“The most effective way to combat desertification and erosion is reforestation […]. That forestry activity is incompatible with environmental objectives is a fallacy. Forests are a source of life. And if forestry activity is conducted appropriately, our quality of life will not only be improved through the economic income that forestry activity will generate, but we will also improve—environmentally speaking—the quality of life of the national population as a whole” (Bluth, 2002, p. 99).

Meanwhile, for the people who spent their childhood in areas dominated by forestry operations, the plantations were a place of recreation and fantasy. One young woman who spent her childhood in the community of Empedrado^8^, and whose father worked in forestry, remembers that he used to take her and her brother “to walk among the pines”, and that:

“we would run between the pines because it was like being in the forest from Little Red Riding Hood. So we would run among the pines. Now and then we'd find spiders, and sometimes other things that were even more terrifying. This is one of my memories as a little girl, running as if through a jungle”^9^.

In another case, a young man who spent his childhood in a house deep in the plantations that surrounded the community of Pellines^10^ remembers that, along with his sister:

“we would spend all day in the forest. We had lots of games; we'd pretend that we could see animals, we'd pretend to be explorers […], as if it were a tropical jungle”^11^.

In fact, there is greater biodiversity hidden by the pine plantations than meets the eye, and this biodiversity forms the basis of the culture of these traditional rural inhabitants, and is passed on in turn to the children. One interviewee remembers that when she was a little girl, she used to go with her mother and brother to the plantations:

“looking for things to eat, things to put in our mouths. In fact, if you turn over a [pine]cone, they drop little fruits that we used to eat […]. We always went to the woods to eat, […] to eat this and that: to eat blackberry, to eat *murtilla*^12^, to eat rosehips, to look for mushrooms to eat, to look for Morchella, to look for *quideñes*^13^ in September. It was all about going to look for food“^14^.

Another interviewee remembers that, as a child, he would go with his mother to the pine woods to collect mushrooms, which they would then dry in the sun on the roof of the family's house before selling. To collect them “we would cross the road toward some hills which I guess belonged to the forestry companies.” For him, this productive activity was a game: “for me it wasn't work. I had a great time exploring the forest.” He also remembers that, after going out collecting mushrooms:

“your hands ended up really stained, for weeks, like with dirt. And you washed your hands but it wouldn't come out. It's from the mushrooms; the mushrooms leave that on your hands. So, it was funny because at that time of year I went to school and there were, I don't know, ten children whose hands were all like that”^15^.

The youngest inhabitants of these regions—who were born many years after the pine and eucalyptus were planted—believe that these are native forests, simply because they have been there “for as long as I can remember”. One person who lived in these forests during their childhood but later moved to Santiago admitted that he only found out “that the pines weren't Chilean” when he was preparing for his university entrance examination:

“Up to *cuarto medio*^16^ I thought that the pines were from here, and that the eucalyptus trees were too. Because they were the trees that I'd grown up with; they're the trees of my childhood. They're the trees I used to climb, where I used to play. So I had no reason to think that the pines weren't from here”^17^.

For these generations, the pines are part of their landscape. They are part of their culture and their identity, and the meaning they acquire is so essential that it can only be formed during childhood. For them, these forests have been there for ever, and are therefore native. They also have all of the cultural attributes of a traditional forest: a place of adventure, a place of danger, and a place to find sustenance.

Similarly, city dwellers, and those people who live in regions further to the north who have never had contact with other types of forest, also believe that these forests are natural. In their eagerness to have some contact with the nature of which they have no knowledge, they place new value on these artificial forests. Many of the plantations were located on the western slopes of the Coastal Range, and with time they became forests with a sea view. Today, this creates a doubly attractive landscape for city people, whose interest has prompted owners of smaller plantations to abandon their original intention of selling their land as timber, deciding instead to market them as landscape. This phenomenon is known as *amenity migration*, which is defined as “migration by people who, having visited a given destination as tourists, decide to return, this time not to visit, but to become inhabitants” (Moss, 2006, p. 3). This leads to the subdivision of appealing plots and the development of real estate and tourism projects which utilize expressive promotional messages such as:

“Privileged place by nature, where the tourist will feel comfortable and quiet, surrounded by an environment of forests, streams and the impressive Maule River (…) “^18^

“Beautiful, appealing plots of land with attractive eucalyptus and pine forest situated in the community of Gualleco, in the district of Curepto. Very close to the sea”^19^

“Urbanized with underground light and drinking water. Paved access, which guarantees its easy and safe arrival in Winter. Land surrounded by beautiful forest that provides the peace and serenity necessary for rest and enjoyment of nature”^20^

In summary, the natural landscape that has been generated in this sector of Chile's Coastal Mountain Range is the result of a mechanism of economic production that sought to recover the profitability and productivity of the land through forestry activity, based on a need to revive the natural attributes of a landscape destroyed by agriculture. The result is a landscape that has gained new value in terms of renewed productive capacity, but which with the passage of time may be re-branded as natural through the favorable image projected by emerging discursive practices.

### Patagonia-aysén: a landscape perceived as purified nature

Patagonia-Aysén is located in southern Chile, and since the nineteenth century, it has been considered as a peripheral zone. It has tended to be associated with the notion of “the end of the world,” an alien place in need of domestication, civilization or integration. This end of the world concept had negative connotations historically, and was seen as something which needed to be overcome in order to achieve modernity, progress or civilization, depending on the context of the time. In fact, even as late as 1920, the area was referred to as “no-man's land” (Bandieri, 2011), a zone that was not truly a part of the nation, or in other words, “empty.” It was for this reason that development initiatives were introduced, focusing on the arrival of transnational livestock enterprises.

This focus on a form of nature that would be both useful to the livestock sector and of benefit to national progress represented a first major step toward an appropriate perception of nature, and, as a result, this type of landscape was promoted for many years. For the most part funded by British money, livestock companies sought to establish themselves between the steppe and the dense forest that dominated the zone, and their operations eventually spread over the entire region to cover more than 110 million square kilometres, or an area almost three times the size of Switzerland. Within this framework of practices and discourses, nature was viewed as a challenge to be overcome, and a real hindrance to the development of the livestock industry. This was true to such an extent that during his visit to the area in 1908, the Swedish explorer Carl Skottsberg made the following comment:

“…The territory is of little importance; only in the eastern section are there good grasses, and moving westward one soon comes up against impenetrable virgin forest” (Martinic, 2014, p. 298).

This discourse adopted by the livestock sector resulted in a relationship of tension which came to define Patagonia-Aysén geographically as an isolated, extreme and distant territory. Public policy implemented in 1930 following the capitalist crisis continued to value this space as a livestock landscape, and lands were made available for colonization. In 1934, the German explorer Max Junge made reference to the delay in incorporating Chilean Patagonia into the ideals of a “modern” and “civilized” country:

“…explorations are beginning once again, this time by air and by land, in an attempt to fill in these empty spaces on the map – the shame of a civilised country – which are still described as ‘unexplored”', adding that “it is estimated that 50% of Western Patagonia is still unknown”, and posing the question: “what is there in these areas? Ice fields, jagged peaks, lakes (…) or can we assume that there are also fertile valleys which have remained overlooked?” (Junge, 1934, p. 29).

Thus the image was formed of a land on the very frontier of civilization, and the process begun during the period emulated the American Wild West: it was a time of colonists whose destiny it was to “extend the fatherland” and build a future amid hostile, complex and adverse nature. In 1942, the German explorer Augusto Grosse wrote that:

“…a colonist with no capital in this Patagonian jungle faces a harsh reality, and faces it alone; as a result he progresses very slowly” (Grosse, 1986, p. 117).

The narrative established around the southern lands associated the idea of progress with the livestock sector. Responsibility for the industry was assigned to the Ministry of Southern Property (created in 1930) under whose supervision the so-called Department of Colonization was formed.

However, in order for the strategy to succeed, clearing and opening up of land was vital. The State became involved in the task, putting into law this process of cleansing. Between 1930 and 1940, the State mandated the opening up of the colonization area by means of a process of “razing”—a practice which had been applied in other places as a means of territorial control—which involved the burning of vast extensions of forest in order to open up or “cleanse” areas for livestock rearing. The contract proposal stated:

“The tenant is in possession of a provisional deed to the land, and must clear at least 100 hectares. If at the end of one year this area has not been cleared, the occupant will not be able to continue toward a Permanent Deed” (AMTC, 1941–1948).

This point of view identified the forest as an obstacle to what was seen as progress. In other words, natural forest represented an undesirable landscape for the purposes of the region's contemporary society.

Thus, for a significant part of the twentieth century, the natural attributes of Patagonia-Aysén were seen in a negative light, and the region's landscape was associated with the notion of savagery. The vision and practices of the State and society at the time gave rise to a landscape of the colonist, of the pioneer or the patriot, striving against hostile and unfavorable nature, but capable of defeating it and thus taking a meaningful step toward the conquest of the territory, and in so doing, strengthening the idea of a national identity based on a “homogeneous dream” or image (Bhabha, 2010).

This landscape of natural forces that needed to be defeated expressed forms and identities rooted in a common geographical imaginary which normalized the view and perception of nature (Deleuze and Guattari, 2002).

In time, the relationship between culture and nature in Patagonia-Aysén came to be seen in a new light (Descola, 2012). Toward the end of the 1980s a new process began to take place. A new interpretive matrix was established in which, this time, nature became the geographical basis of a social identity, creating the idea of a “*Reserva de Vida*” (Life Reserve), an official title currently applied to the region (Gobierno Regional de Aysén, 2009). The approach now specifies nature as a “requirement” of the State and public policy, but this time in order to purify it [(Jazeel, 2014), p. 79].

This new identity is based on an emphasis of the natural world as representing the highest values and wealth potential, in a context in which nature (an expression which previously represented savagery) is the basic condition for environmental sustainability, which itself has become an essential requirement of development. Thus, a series of practices and actions are promoted, generating new imaginaries of development and changes in relations with the regional environment (Nuñez et al., 2014a; Mellado, 2015).

The new discourse of nature in Patagonia-Aysén, symbolized by the slogan “*Aysén, Reserva de Vida*”^21^, now replaces old social rationalities and communicates fresh messages: biodiversity, pristine wilderness, the purity of distance, the advantage of isolation from the world. This is a new environmental rationality which shows that, in the words of Leff:

“biodiversity comprises not only a multiplicity of forms of life, but complete nature reserves – biologically and culturally diverse territories and habitats – which are gaining value due to their genetic richness, their ecotourism resources, and their function as carbon fixers” (Leff, 2004, p. 113).

**Graph 3:**
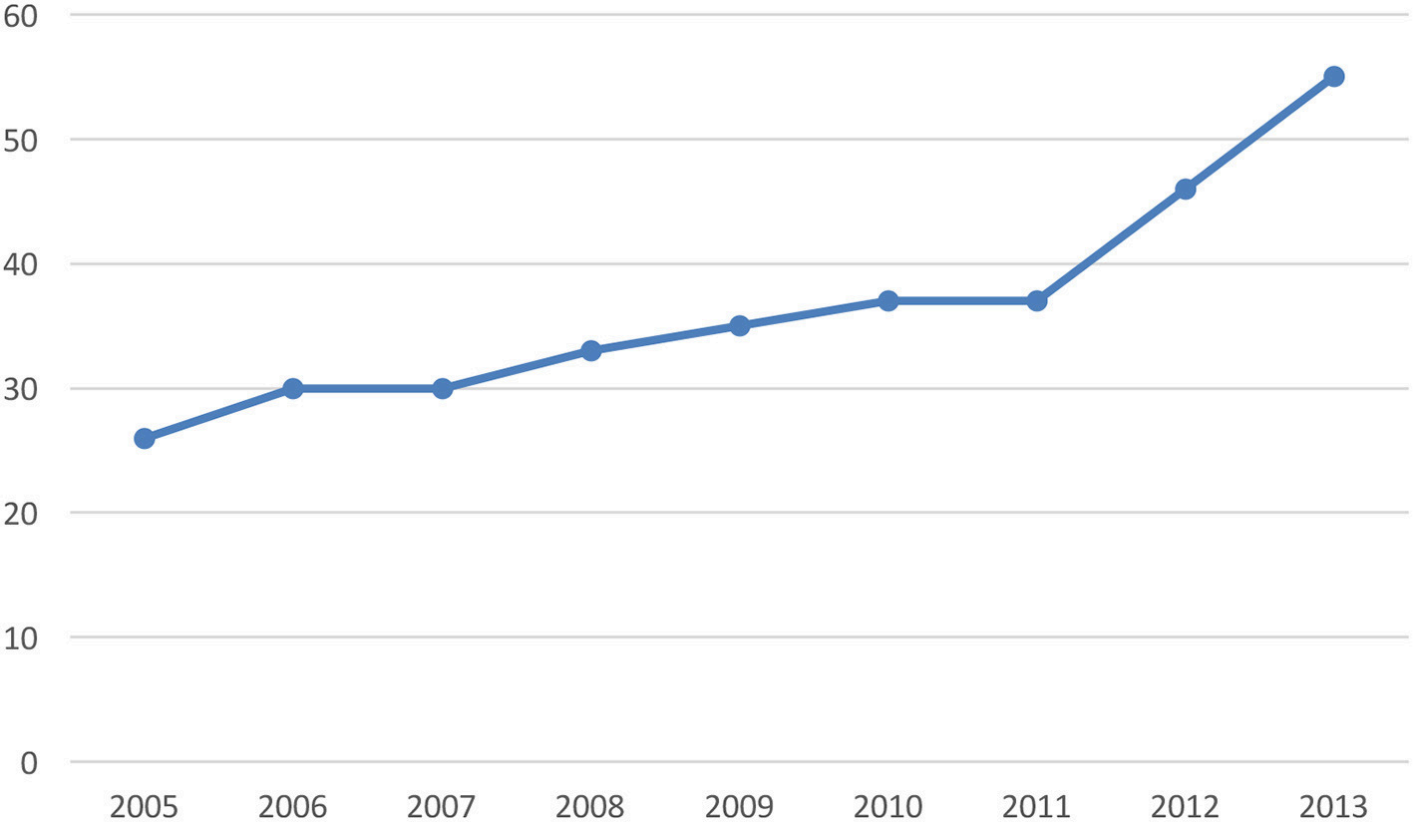
Numbers of companies of tourism services. Activities of travel agencies, organizers of trips, assistance to tourists. Source: Tourism Chilean National Agency (Subsecretaría de Turismo), Ministry of Economy, Promotion and Tourism. http://www.subturismo.gob.cl/documentos/estadisticas/estadisticas-regionales.

**Graph 4:**
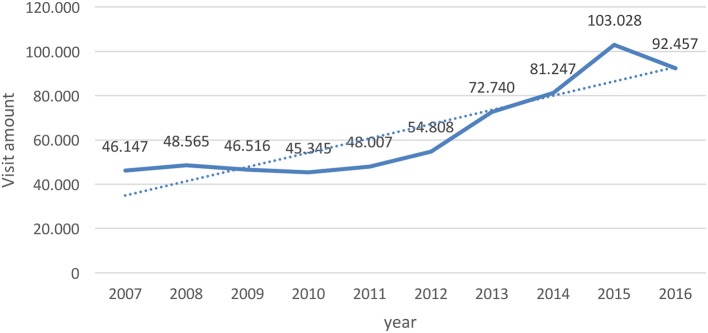
Amount of visits to protected areas in Aysen Region (natural reserves). Source: Tourism Chilean National Agency (Subsecretaría de Turismo), Ministry of Economy, Promotion and Tourism. http://www.subturismo.gob.cl/documentos/estadisticas/estadisticas-regionales.

From the begin of the XXI century, we can observe a new field of business coming from green ideas, sustainable development, eco-tourism, etc. This is reflected in the increase of services linked with tourism in the region (see Graphs 3 and 4).

Ultimately, “new” and “greened-up” nature in Patagonia-Aysén has become a utopian discourse of the type described by Foucault:

“Utopias are emplacements having no real place. They are emplacements that maintain a general relation of direct or inverse analogy with the real space of society. They are society perfected or the reverse of society, but in any case these utopias are spaces that are fundamentally and essentially unreal” (Foucault, 2010, p. 69).

In other words, an independently existing nature—complete with its own rationality—is installed, whose stable, harmonious, ethical and universal purpose is to serve as a support for a globalized humanity on its journey to a new destiny.

The protection of forest previously viewed as worthless and useless (branded *monte* (bush), *mala hierba* (weeds), and *maleza* (scrub) by pioneering colonizers, and whose burning or razing was enshrined in law by the State in the mid-twentieth century) was no longer incompatible with sustainable development and its global impact (Nuñez et al., 2017).

On this global scale, Patagonia-Aysén has over recent decades gained positive value as “the end of the world”. One very visible impact is the sustained increase in the concentration of land in the hands of new investors interested in a kind of “green capitalism” (Ayala and Moritz, 2012). The fundamental attraction of these areas today is the environmental value of natural landscapes. This has triggered a rapid process of sale and purchase of land through which the traditional colonists are now beginning to move out due to unexpected demand for lands which, according to their traditional livestock rearing logic, hold no value whatsoever. According to one testimony:

“My lands were worth little, because they were full of forests, lakes and rivers…I couldn't do much there. Of my 1,200 hectares I could use only 100 for my cows. So I was happy to sell” (colonist, Cochrane, over 60 years old).

This process of land transfer reveals a tendency already present in central Chile: the same business groups that have investments in the central and northern part of the country—principally in the mining, forestry, infrastructure and energy sectors, operations which have come under scrutiny due to their environmental impact—are beginning to acquire property in the south based on the high value of natural landscapes. The concentration of “nature-rich” property becomes a vital aspect of a strengthening global environmentalist-capitalist discourse, and Patagonia-Aysén is one of the most iconic cases worldwide.

**Figure 7:**
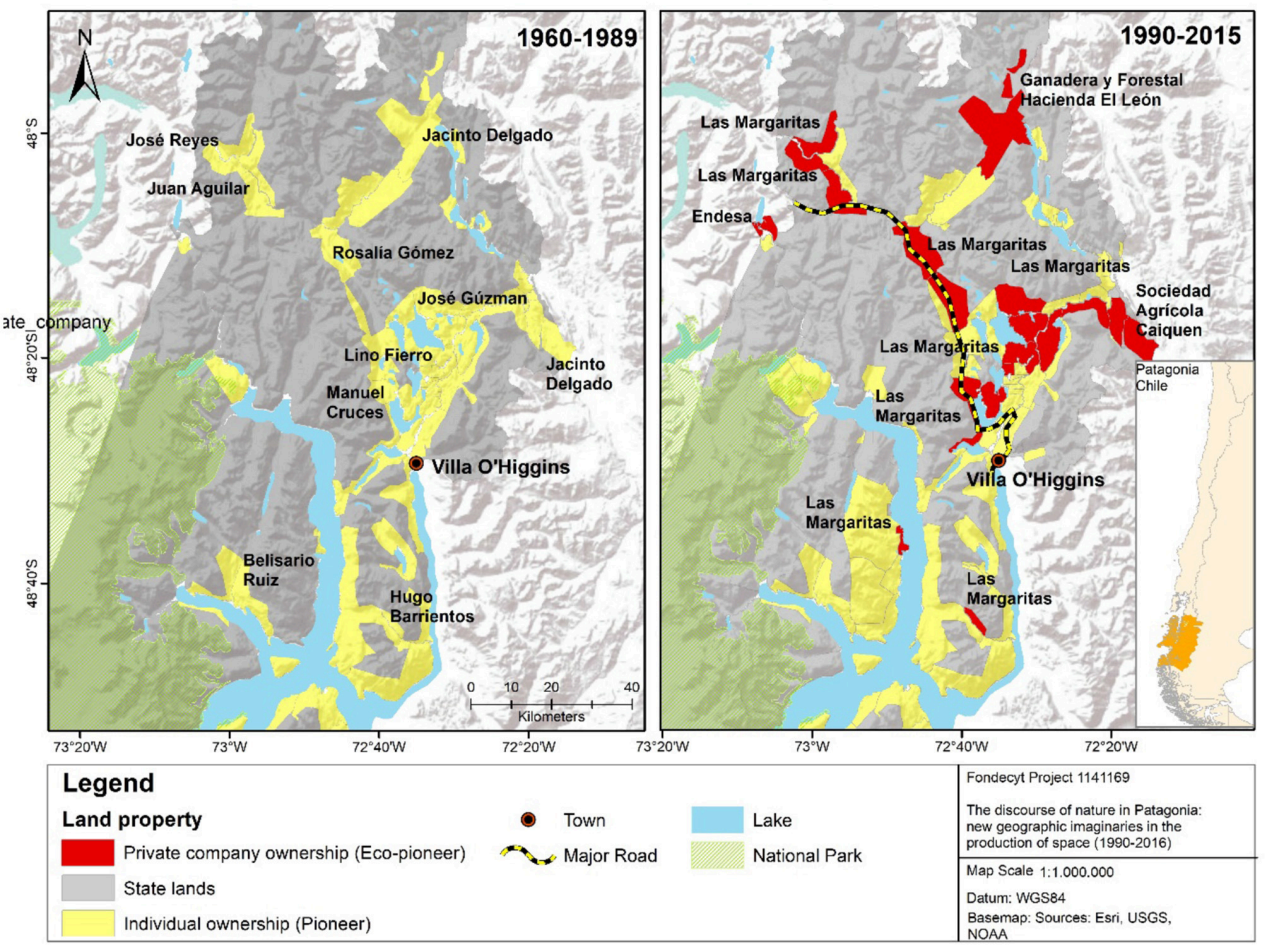
The “nature-rich” property process in Villa O'Higgins. Source: Compiled by the authors.

Figure 7 provides an illustration of this process. It shows the district of O'Higgins, in the south of the region, and the concentration in 2015 of property owned by numerous groups, in particular Las Margaritas S.A., a conservationist company controlled by the Luksic Group. The latter is one of wealthiest corporations in Chile and worldwide, and owner of a series of extractive operations, generally mining, all over the planet (Nuñez et al., 2014b).

Nature, now purified and transformed into a vital system, is given meaning by a green imaginary full of biodiversity and founded on an ideal: the perfect balance between the activities of man and his environment, and from which man's new destiny is forged. In the words of Swyngedouw:

“Normative power inscribed in Nature is invoked as an organizing principle that is transcendental and universal, allegedly residing outside the remit allocated to humans and non-humans alike but that exercises an inescapable performative effect and leaves a non-alienable imprint. This is a view that sees Nature as something given, as a solid foundational (or ontological) basis from which we act and that can be invoked to provide an anchor for ethical or normative judgements of ecological, social, cultural, political, or economic procedures and practices” (Swyngedouw, 2015, p. 17).

This new ontological support provided by nature justifies the proliferation of a new conservationist real estate business, and, in parallel, sustains the development of large-scale ecotourism projects. This process is not only notoriously selective and exclusive, but invokes the need to place nature in the fixed, static and objective world: “forest preserved in its natural form,” “natural landscapes unique to Chile,” “unexplored area of pristine native forests,” “unique place which is re-writing the history of Aysén's unexplored forests and rivers…it offers the chance to experience and live nature in its wildest form, in a privileged location which was, until only recently, completely inaccessible”^22^ Thus, everything is presented as starting anew, and the visitor is invited to take part in the re-foundation of a new natural world.

It is clear that the current geographical imaginary of Patagonia-Aysén as a *Reserva de Vida* has not been created based on the inherent or “natural” characteristics of the area, but rather is the result of a process of territorial production in which the relationship between culture and nature develops as a co-fabrication or co-belonging between the subject and the landscape which he himself constructs. Thus, appreciation of the value of nature in a globalized context represents simply a new geographical colonization of spaces on the nation's periphery.

## Conclusion: how should we perceive chile's new natural landscapes?

The historical and spatial journey that has defined Chile's landscape goes hand in hand with the creation of imaginaries and representations. As can be seen from the cases covered in the present work, a number of paths have converged such that, by the beginning of the twenty-firstt century, discourses concerning the value of nature were beginning to gain relevance in everyday life. Notions to do with the concept of “vocational” land use, whether with reference to the forestry sector, livestock rearing or conservationism, are closely associated with a projected idea of natural landscape which at various points in social and cultural history has been given different emphasis.

No more than 40 years on from the beginning of the forestry boom, and more than 80 years since the first significant forestation campaigns were launched, monoculture forestry has erased the agricultural landscape of the Coastal Mountain Range from all memory. Younger inhabitants of central Chile believe these commercial forestry plantations to be natural forests, given that they have been there “for as long as I can remember”^23^. These inhabitants are living testimony of the nature that they have experienced, and give sense to what has become the natural landscape of the forestry plantations. This becomes more widespread with the passage of time, and is further ingrained by current practices based on amenities which today make an ever growing contribution to the land market.

Meanwhile, in Patagonia-Aysén, the whole of the twentieth century reflected the national view that this was an isolated, unincorporated and absent territory, characterized by a wild, hostile and uncivilized landscape which needed to be conquered. It could be said that this was an undesired natural landscape. Around 1990, Patagonia-Aysén was swept up in a discursive process of global proportions which would turn out to be vital in justifying its status of exceptionality or periphery. This time, however, it strengthened appreciation of a nature which is also global (as a *Reserva de Vida*), and in doing so, this undesirable landscape is becoming a highly valued commodity, precisely for its natural state.

However, considering all of the background and processes covered in the present work, one element stands out as being critical and worthy of discussion in terms of the way in which landscape—and, in particular, natural landscape—is perceived. While on one hand the concept of nature has been key to creating conditions which during the early twenty-first century have led to the definition of what is today known as environmentally sustainable development, it can also paradoxically be observed that the same argument, posed in a context of discursive production which draws on the idea of what is natural, has been the foundation of a new form of market in territorial production. The result has been the expansion of the forestry sector and its associated socio-environmental consequences for central Chile, while in Patagonia-Aysén the result has been a transformation in occupation and the concentration of land ownership.

In both cases, the concepts of natural landscape, vocation and nature have accentuated processes of territorial production, putting stress on the notion of environmentally sustainable development and presenting the questions: Who benefits from the idea of and the argument surrounding the importance of nature? What processes are triggered by the idea of natural landscapes?

In the context of extreme neoliberal economies like Chile, discourses which drive the need for or desirability of natural landscapes appear in fact to accentuate inequality and lead to a distancing from the very objectives of environmentally sustainable development. Thus, the paradox of discourses of nature is that, while providing a basis for bringing about changes which help things to stay the same, they simultaneously generate new inequalities in terms of access to these natural landscapes.

## Author contributions

EA: Corresponding author; in charge of theoretical aspects, revision and guide of the discussion. MF: In charge of Cordillera de la Costa elements, representation and historical elements about the forested lands in Central Chile. Statistical analysis, debates, etc. AN: In charge of elements of the Patagonia-Aysén description and analysis, included discussions and debates about it.

### Conflict of interest statement

The authors declare that the research was conducted in the absence of any commercial or financial relationships that could be construed as a potential conflict of interest. The reviewer IH and handling editor declared their shared affiliation at the time of review.

## Footnotes

^1^The study area does not include the La Araucanía Region, or the southern part of the Province of Arauco. It is within this zone that the Mapuche population is most concentrated, and is referred to by some as “the Mapuche territory.” The region is covered by extensive commercial forests, but the presence of this ethnic group makes the process of forestry expansion completely different in nature compared with other areas of forestry plantations.

^2^“Editorial”. *Revista Forestal Chilena*. Tomo II. N. 11, March 1952, p. 293.

^3^“Wood. Its production and markets”, *Chile Maderero*, N. 4, June-July 1954, p. 38.

^4^“Speech made by the President of the Chilean Timber Corporation”. *Chile Maderero*, N. 4, July-August 1956, p. 12.

^5^Decree Law N. 656, “Forests Law”, 06-11-1925, Art. 4.

^6^This includes the provinces of Maule, Linares, Ñuble, Biobío, Concepción, Arauco, Malleco and Cautín. Source: INFOR (1966).

^7^See Hartwig, 1994; Corporación Chilena de la Madera, 1999; Bluth, 2002.

^8^A district situated in the Maule region, in the heart of the local forestry plantations.

^9^Former resident of the district of Empedrado, 26 years of age, interviewed 19/04/2017.

^10^Part of the district of Constitución, also in the Maule region.

^11^Former resident of Pellines, 23 years of age, interviewed 14/04/2017.

^12^Chilean guava.

^13^Cyttaria espinosae.

^14^Former resident of the district of Empedrado, 26 years of age, interviewed 19/04/2017.

^15^Former resident of Pellines, 23 years of age, interviewed 14/04/2017.

^16^The final year of secondary education in Chile.

^17^Former resident of Pellines, 23 years of age, interviewed 14/04/2017.

^18^“La Palmilla” tourist place” [http://www.lapalmilla.cl/].

^19^“Loteo La Mina, Gualleco, Curepto” [http://www.vivastreet.cl/loteos-lotes/talca-provincia/loteo-la-mina-gualleco–curepto—-sector-costa-del-maule-/144823426].

^20^“Condominio Fundo Santo Domingo, Pichilemu” (Santo Domingo real estate project, Pichilemu) [http://www.ingeocad.cl/?portfolio=proyecto-en-providencia].

^21^This slogan it was made by the Regional Government, in the context of the Regional Strategy for Development of the Aysén Region (2008). From there, the slogan has an extended acceptation in all stakeholders.

^22^For example, http://www.chileboutique.cl/chileboutique/program/Puyuhuapi%20Lodge%20&%20Spa%20Media%20Pensi%C3%B3n.

^23^It is worth nothing that this phenomenon of naturalisation does not apply to all of the forested territories. In forested areas within what may be referred to as “the Mapuche territory”, this naturalisation process has not occurred. On the contrary, these areas manifest a firm rejection of monoculture forestry. This rejection is based on a number of factors, the primary being the historical claim made by the Mapuche people to lands usurped from them. Those lands and that territory to which they lay claim today currently lie in the hands of forestry companies (Correa et al., 2005; Correa and Mella, 2010).
